# Editorial: Respiratory diseases in veterinary medicine: Time for some fresh air

**DOI:** 10.3389/fvets.2022.1033768

**Published:** 2022-10-04

**Authors:** Mayara Fernanda Maggioli, Fernando Viçosa Bauermann, Ana Paula Junqueira-Kipnis

**Affiliations:** ^1^Veterinary Virology Laboratory, Department of Veterinary Pathobiology, College of Veterinary Medicine, Oklahoma State University, Stillwater, OK, United States; ^2^Department of Biosciences and Technology, Institute of Tropical Pathology and Public Health, Federal University of Goiás, Goiânia, Goiás, Brazil

**Keywords:** bovine respiratory disease, swine, kennel cough, coronavirus, rotavirus, mycoplasma, Bordetella

Respiratory diseases are a major cause of illness in veterinary medicine, leading to extensive economic losses, especially in animal production settings, with detrimental consequences to animal health and welfare (1–5). Infections by bacterial agents require the use of antibiotics, raising concerns about antimicrobial resistance risks for animal and human health (6, 7). Respiratory diseases are complex and multi-layered. Infection may involve a single agent or a myriad of pathogens, and both the host and environmental factors play a role in disease occurrence, severity, and outcome. This Research Topic is all-encompassing in order to bring light (or a breath of fresh air, if you will) to this complex subject, covering many aspects of respiratory disease in veterinary science including host-pathogen interaction, the impact of respiratory diseases on work animals, disease prevention, and immunity.

Bovine Coronavirus (BCoV) has long been identified as a causative agent of cattle diarrhea. However, its role as a respiratory pathogen is less clear. Frucchi et al., report that BCoV, as well as *Pasteurella multocida* and *Mannheimia haemolytica*, were frequently detected in nasal swabs samples from heifer calves in high-production dairy herds in Brazil, suggesting a role of BCoV in Bovine respiratory disease complex (BRDC). Molecular characterization of BCoV N and S1 genes in swab samples revealed that the detected strains are ancestrally different from strains previously reported in the region. The high frequency of BCoV (56%) detection and the identification of a different variant circulating in Brazil suggest that BCoV may be a major BRDC pathogen among heifer calves. Corroborating with this finding, Soules et al. experimentally infected calves intranasally with virulent BCoV. The detection of BCoV in bronchoalveolar lavage, nasal turbinate, and trachea along with histopathologic lesions in the upper and lower respiratory tract further implicates BCoV plays a role in respiratory disease in cattle.

Rotavirus (RV) is also known to cause acute gastroenteritis worldwide, affecting various species, including pigs, cattle, poultry, and humans. While epidemiological studies report respiratory symptoms concurrently with fecal and nasal shedding, RV respiratory infections have scarcely been investigated. Nelsen et al. at SDSU screened swine lung clinical samples submitted to the diagnostic laboratory for the presence of porcine rotavirus A (RVA) genome by quantitative reverse transcription PCR (qRT-PCR). RV was detected in 30.8% (22 samples) of lung samples obtained from conventionally reared pigs with respiratory signs. *In situ* hybridization (ISH) showed that RV genome localization in positive lung samples was restricted to alveolar and interstitial macrophages and bronchiolar epithelial cells. Analysis of 120 archival formalin-fixed and paraffin-embedded lung samples by ISH revealed another 10 RV-positive cases with similar RV genome localization patterns.

*Mycoplasma hyopneumoniae* is another relevant swine respiratory pathogen. This bacterium is the primary agent in the swine enzootic pneumonia infectious and plays a role in the swine respiratory disease complex (SRDC) with significant economic losses to the swine industry worldwide. Despite its impact, control and prevention are challenging due to the limited characterization of the antigenic repertoire, virulence profile, and resistance to antimicrobials. Additional complicating factors regarding *M. hyopneumoniae* epidemiology are distinct circulating strains and the intrinsic resistance against β-lactam antibiotics, sulfonamides, and trimethoprim. A few reports have shown acquired antimicrobial resistance against some antibiotics and associated resistance mechanisms. However, a clear picture of the virulence and pathogenicity of *M. hyopneumoniae* is still missing, and the potential impact of strain variability on disease severity is likewise not always well-defined. Therefore, the identification and characterization of *M. hyopneumoniae* strains circulating within a population or geographical region are essential. In the current topic, Zong et al. evaluated the growth and morphology, pathogenesis, and antimicrobial sensitivity characteristics of an *M. hyopneumoniae* strain isolated from Chinese native Enshi black pig lungs (*M. hyopneumoniae* strain ES-2). The study also identified 2 genes only present in pathogenic *M. hyopneumonia*e strains, with great potential as a virulence marker and a tool in clinical diagnosis.

Although usually considered pets, dogs are frequently raised as working animals. Dogs serve, for example, as guides for vision-impaired owners, livestock guardian dogs, and as scent detectors. Canine olfactory detection has a broad range of purposes, including detection of drugs and explosives, medical detection, or search and rescue (8–12). No different from other animals, dogs raised in groups are at higher risk of respiratory infections (13). *Bordetella bronchiseptica* is responsible for severe respiratory disease in dogs, swine, and rabbits and is a significant contributor to canine infectious tracheobronchitis (also known as “kennel cough”). While upper respiratory infections and their usual clinical signs can have obvious implication for the ability of these dogs to carry out their scent detection duties, the potential impact of the intranasal anti-Bordetella vaccine administration on the olfactory capabilities of dogs have not been evaluated. The delivery to the nasal mucosa epithelium elicits a local immune response, and this localized inflammation could cause hyposmia/anosmia, hampering the dog's ability to conduct detection work safely and effectively. Collins et al. evaluated different anti-Bordetella vaccine regiments, including oral and intra-nasal vaccine delivery. Luckily, odor thresholds were not influenced by any vaccine strategy. Single intranasal or oral Bordetella vaccine did not impact detection by dogs, while prime with an oral vaccine followed by intranasal boost led to a slight increase in time to detection but not the ability to detect the target odor.

Studies included in this Research Topic portrayed the ever-evolving nature of respiratory infectious diseases, especially under intensive production settings that favor transmission and spread. This Research Topic aimed to highlight and promote discussion regarding respiratory diseases in veterinary science. The complied research portraited the investigation of new pathogens, but mostly ways to re-think old pathogens and their role in newly identified problems. It is noteworthy that detection, treatment, and prevention of respiratory diseases in animals is not only limited to protecting animal health and welfare but also supports food security and has a profound positive impact on human health.

## Author contributions

MM, FB, and AJ-K wrote the editorial. All authors contributed to the article and reviewed and approved the submitted version.

## Conflict of interest

The authors declare that the research was conducted in the absence of any commercial or financial relationships that could be construed as a potential conflict of interest.

## Publisher's note

All claims expressed in this article are solely those of the authors and do not necessarily represent those of their affiliated organizations, or those of the publisher, the editors and the reviewers. Any product that may be evaluated in this article, or claim that may be made by its manufacturer, is not guaranteed or endorsed by the publisher.

## References

[1] Dubrovsky SA van Eenennaam AL Aly SS Karle BM Rossitto PV Overton MW. Preweaning cost of bovine respiratory disease (BRD) and cost-benefit of implementation of preventative measures in calves on California dairies: the BRD 10K study. J Dairy Sci. (2020) 103:1583–97. 10.3168/jds.2018-15501 31759608

[2] Blakebrough-Hall C McMeniman JP González LA. An evaluation of the economic effects of bovine respiratory disease on animal performance, carcass traits, and economic outcomes in feedlot cattle defined using four BRD diagnosis methods. J Anim Sci. (2020) 98:skaa005. 10.1093/jas/skaa005 31930299 PMC6996507

[3] Gebhardt JT Tokach MD Dritz SS DeRouchey JM Woodworth JC Goodband RD. Postweaning mortality in commercial swine production II: review of infectious contributing factors. Transl Anim Sci. (2020) 4:485–506. 10.1093/tas/txaa052 32705048 PMC7277696

[4] Saade G Deblanc C Bougon J Marois-Créhan C Fablet C Auray G. Coinfections and their molecular consequences in the porcine respiratory tract. Vet Res. (2020) 51:1–19. 10.1186/s13567-020-00807-8 32546263 PMC7296899

[5] J. R, A. G, E. D. Prevalence of canine infectious respiratory disease complex pathogens in dogs in georgia and north carolina. J Vet Intern Med. (2016) 30:572–6.

[6] Stanford K Zaheer R Klima C McAllister T Peters D Niu YD. Antimicrobial resistance in members of the bacterial bovine respiratory disease complex isolated from lung tissue of cattle mortalities managed with or without the use of antimicrobials. Microorganisms. (2020) 8:288. 10.3390/microorganisms8020288 32093326 PMC7074851

[7] Guardabassi L Apley M Olsen JE Toutain PL Weese S. Optimization of antimicrobial treatment to minimize resistance selection. Microbiol Spectr. (2018) 6. 10.1128/microbiolspec.ARBA-0018-2017 29932044 PMC11633575

[8] Lazarowski L Dorman DC. Explosives detection by military working dogs: olfactory generalization from components to mixtures. Appl Anim Behav Sci. (2014) 151:84–93. 10.1016/j.applanim.2013.11.010

[9] Rust LT Nizio KD Wand MP Forbes SL. Investigating the detection limits of scent-detection dogs to residual blood odour on clothing. Forensic Chem. (2018) 9:62–75. 10.1016/j.forc.2018.05.002

[10] Chambers CL Vojta CD Mering ED Davenport B. Efficacy of scent-detection dogs for locating bat roosts in trees and snags. Wildl Soc Bull. (2015) 39:780–7. 10.1002/wsb.598

[11] Dickey T Junqueira H. Toward the use of medical scent detection dogs for COVID-19 screening. J Am Osteopathic Assoc. (2021) 121:141–8. 10.1515/jom-2020-0222 33567089

[12] Jendrny P Twele F Meller S Osterhaus ADME Schalke E Volk HA. Canine olfactory detection and its relevance to medical detection. BMC Infect Dis. (2021) 21:1–15. 10.1186/s12879-021-06523-8 34412582 PMC8375464

[13] Reagan KL Sykes JE. Canine infectious respiratory disease. Vet Clin North Am Small Anim Prac. (2020) 50:405–18. 10.1016/j.cvsm.2019.10.009 31813556 PMC7132485

